# Effect of Biogenic Silver Nanoparticles on the Quorum-Sensing System of *Pseudomonas aeruginosa* PAO1 and PA14

**DOI:** 10.3390/microorganisms10091755

**Published:** 2022-08-30

**Authors:** Erika Kushikawa Saeki, Heloísa Moreira Martins, Larissa Ciappina de Camargo, Laís Anversa, Eliandro Reis Tavares, Sueli Fumie Yamada-Ogatta, Lucy Megumi Yamauchi Lioni, Renata Katsuko Takayama Kobayashi, Gerson Nakazato

**Affiliations:** 1 Regional Laboratory Center, Adolfo Lutz Institute, Presidente Prudente 19013-050, SP, Brazil; 2 Laboratory of Basic and Applied Bacteriology, Department of Microbiology, Biological Sciences Center, State University of Londrina, Londrina 86057-970, PR, Brazil; 3 Regional Laboratory Center, Adolfo Lutz Institute, Bauru 17015-110, SP, Brazil; 4 Laboratory of Molecular Biology of Microorganisms, Department of Microbiology, Biological Sciences Center, State University of Londrina, Londrina 86057-970, PR, Brazil

**Keywords:** AgNPs, antivirulence, gene regulation, quorum quenching

## Abstract

The increase in multidrug-resistant microorganisms represents a global threat requiring the development novel strategies to fight bacterial infection. This study aimed to assess the effect of silver nanoparticles (bio-AgNPs) on bacterial growth, biofilm formation, production of virulence factors, and expression of genes related to the quorum-sensing (QS) system of *P. aeruginosa* PAO1 and PA14. Biofilm formation and virulence assays were performed with bio-AgNPs. RT-qPCR was carried out to determine the effect of bio-AgNPs on the QS regulatory genes *lasI, lasR, rhlI, rhlR, pqsA*, and *mvfR*. Bio-AgNPs had an MIC value of 62.50 μM, for both strains. Phenotypic and genotypic assays were carried out using sub-MIC values. Experimental results showed that treatment with sub-MICs of bio-AgNPs reduced (*p* < 0.05) the motility and rhamnolipids and elastase production in *P. aeruginosa* PAO1. In PA14, bio-AgNPs stimulated swarming and twitching motilities as well as biofilm formation and elastase and pyocyanin production. Bio-AgNP treatment increased (*p* < 0.05) the expression of QS genes in PAO1 and PA14. Despite the different phenotypic behaviors in both strains, both showed an increase in the expression of QS genes. Demonstrating that the bio-AgNPs acted in the induction of regulation. The possible mechanism underlying the action of bio-AgNPs involves the induction of the rhl and/or pqs system of PAO1 and of the las and/or pqs system of PA14. These results suggest that exposure to low concentrations of bio-AgNPs may promote the expression of QS regulatory genes in *P. aeruginosa*, consequently inducing the production of virulence factors such as elastase, pyocyanin, and biofilms.

## 1. Introduction

*Pseudomonas aeruginosa* is a Gram-negative, mobile, aerobic, non-spore-forming bacterium. Due to its versatile metabolism, *P. aeruginosa* can grow in aquatic environments and in soil, causing infections in plants and animals and a variety of diseases in humans, particularly in immunocompromised individuals [1,2,3].

The World Health Organization recognized the difficulty in treating *P. aeruginosa* infections and included the bacterium in its priority list of pathogens for which new drugs are urgently required, particularly for the treatment of carbapenem-resistant isolates [4], which are considered a major threat to public health.

Compounds with antivirulence activity, that is, those that can control pathogens without exerting bacteriostatic or bactericidal action, have been proposed as potential therapeutic agents [5,6]. The quorum-sensing (QS) system can be affected by attenuation of QS communication, disruption of receptor proteins, degradation of autoinducing signals, or inhibition of the synthesis of signaling molecules [7,8]. The QS system is a regulatory center of virulence factors and has been widely studied as a target for antivirulence therapy [6,9].

QS is a mechanism of cellular communication between bacteria mediated by the secretion of extracellular signaling molecules (autoinducers). The concentration of autoinducers depends on microbial density. In general, the system consists of an enzyme that catalyzes the synthesis of signaling molecules and a receptor that binds to the signaling molecule and induces the expression of genes responsible for various physiological mechanisms, such as cell density and virulence factor production [10,11]. Chemical communication through the QS system is a central feature of bacterial life, allowing bacteria to remain in a community and defining collective behaviors [12].

The QS system of *P. aeruginosa* consists of three hierarchically organized systems, namely las, rhl, and PQS [13,14,15]. Due to the importance of QS to bacterial pathogenicity, many studies have focused on QS interference. Metal nanoparticles have been identified as potent agents against QS [16].

Studies on the QS system of *P. aeruginosa* and its regulation are of great importance to better understand the response of bacteria to metal nanoparticles. For this, it is necessary to identify the mechanisms involved in bacterial pathogenicity. This study aimed to investigate the effect of biogenic silver nanoparticles (bio-AgNPs) on *P. aeruginosa* growth, virulence factors, biofilm formation, and QS.

## 2. Materials and Methods

### 2.1. Bacterial Strains and Culture Conditions

The reference strains *P. aeruginosa* PAO1 and UCBPP-PA14 (PA14) were kindly provided by Regina Lúcia Baldini (University of São Paulo) and Laurence G. Rahme (Harvard University). Stock cultures were kept at −80 °C in Luria–Bertani (LB) broth (Neogen, Lansing, MI, USA) containing 20% (*v*/*v*) glycerol (Synth, Diadema, SP, Brazil).

### 2.2. Bio-AgNPs

Bio-AgNPs were synthesized by reduction of silver nitrate catalyzed by a cell-free enzyme preparation from fungus *Fusarium oxysporum* (strain 551). The fungal inoculum was obtained from the Laboratory of Molecular Genetics, University of São Paulo, Piracicaba, São Paulo State, Brazil. *F. oxysporum* was grown for 7 days at 28 °C on 0.5% *w*/*v* yeast extract (Neogen, USA), 2% *w*/*v* malt extract (Neogen, USA), 2% *w*/*v* agar (Acumedia, Lansing, MI, USA), and distilled water. Then, the fungal biomass (0.1 g/mL) was mixed with sterile distilled water and incubated at 28 °C for 72 h under stirring (150 rpm). Next, the cell-free filtrate was mixed with 0.01 M silver nitrate (AgNO_3_, Sigma–Aldrich, Steinheim am Albuch, Germany ) and incubated for 15 days at 28 °C in the dark. Finally, Bio-AgNPs were washed with distilled water, centrifuged at 27,000× *g* and 4 °C for 30 min, and incubated in an ultrasonic bath for 30 min. Washing steps were repeated three times [17].

These bio-AgNPs have been synthesized, quantified, and characterized by our research group and these results have been previously published [18]. According to Scandorieiro et al. [18], the nanoparticle used in this study exhibited a plasmonic absorption band in the visible region of the spectrum (near 420 nm), spherical shape, and an average size and zeta potential of 73.1 ± 0.5 nm and −24.2 ± 2.1 mV, respectively.

### 2.3. Minimum Inhibitory Concentration (MIC)

The MIC of bio-AgNPs for *P. aeruginosa* PAO1 and PA14 was determined by the microdilution method in 96-well plates [19]. For that, bio-AgNps were diluted in Mueller–Hinton broth (MHB, Difco, Sparks, MD, USA) to concentrations ranging from 7.81 to 1000 µM. The plates were incubated at 37 °C for 24 h. Positive (*P. aeruginosa* grown in the absence of bio-AgNPs) and negative (bio-AgNPs or MHB alone) controls were performed. All experiments were conducted in triplicate and repeated at least three times.

### 2.4. Growth Curve Analysis

The effect of bio-AgNPs on the growth kinetics of *P. aeruginosa* PAO1 and PA14 was assessed by the plating method, as described by Jorgensen [20]. Bacterial inoculum was cultivated in LB broth (Neogen, Lansing, MI, USA) with bio-AgNPs at concentrations of 1/2, 1/4, and 1/8 MIC at 37 °C for 48 h. A control growth curve was constructed by incubating bacterial strains in the absence of bio-AgNPs. Bacterial growth was analyzed at six incubation times (0, 4, 8, 24, 32, and 48 h).

### 2.5. Assessment of Virulence Factors

#### 2.5.1. Biofilm Formation

Biofilm formation capacity was analyzed in 96-well polystyrene plates using the crystal violet method described by Ramos-Vivas et al. [21], with some modifications (Supplementary Table S1). Bacterial isolates were cultured on LB agar (Neogen, Lansing, MI, USA) at 37 °C for 24 h. Then, 180 µL of LB broth (Neogen, Lansing, MI, USA) and 20 µL of *P. aeruginosa* inoculum (initial concentration of 1.5 × 10^6^ CFU/mL) were added to each well and incubated at 37 °C for 24 h in the presence (1/2, 1/4, and 1/8 MIC) and absence (control) of bio-AgNPs. The supernatant of each well was discarded, and the cell layer was washed three times with phosphate buffer solution (PBS, pH 7.2), fixed with 250 µL of absolute methanol PA (Merck, Darmstadt, Germany) for 10 min, and stained with a 1.0% *w*/*v* aqueous solution of crystal violet (Merck, Darmstadt, Germany) for 15 min. Subsequently, the dye solution was discarded, and each well was washed three times with ultrapure water and treated with 250 µL of 33% *v*/*v* glacial acetic acid (Merck, Darmstadt, Germany). Assays were conducted in the presence (1/2, 1/4, and 1/8 MIC) and absence of bio-AgNPs. Absorbance readings were taken at 620 nm on a spectrophotometer (Multiskan FC, Thermo Scientific, Waltham, MA, USA). Experiments were repeated four times per isolate, and results are presented as mean and standard deviation.

#### 2.5.2. Swarming Motility

*P. aeruginosa* isolates were grown in LB broth (Neogen, Lansing, MI, USA) for 24 h at 30 °C. Ten microliters of a *P. aeruginosa* suspension containing 10^8^ CFU/mL in the presence (1/2, 1/4, and 1/8 MIC) or absence (control) of subinhibitory concentrations of bio-AgNPs were inoculated at the center of swarming agar plates containing 1% *w*/*v* glucose (Synth, Diadema, SP, Brazil), 0.5% *w*/*v* peptone (Acumedia, Lansing, MI, USA), 0.2% *w*/*v* yeast extract (BD, Sparks, MD, USA), and 0.5% *w*/*v* agar (BD, Sparks, MD, USA). Plates were incubated without inversion for 24 h at 30 °C [22].

#### 2.5.3. Swimming Motility

*P. aeruginosa* isolates were seeded on LB agar (Neogen, Lansing, MI, USA) at 37 °C for 24 h. Swimming agar plates containing 1.0% *w*/*v* tryptone (Acumedia, Lansing, MI, USA), 0.5% *w*/*v* sodium chloride (Merck, Darmstadt, Germany), and 0.3% *w*/*v* agar (BD, Sparks, MD, USA), previously equilibrated to room temperature, were inoculated on the surface with one colony in the presence (1/2, 1/4, and 1/8 MIC) or absence (control) of subinhibitory concentrations. Plates were incubated without inversion for 24 h at 30 °C [23].

#### 2.5.4. Twitching Motility

*P. aeruginosa* isolates were seeded on LB agar (Neogen, Lansing, MI, USA) and incubated at 37 °C for 24 h. One colony of each isolate was inoculated in the presence (1/2, 1/4, and 1/8 MIC) or absence (control) of subinhibitory concentrations of bio-AgNPs at the bottom of twitching agar plates containing 1.0% *w*/*v* tryptone (Acumedia, Lansing, MI, USA), 0.5% *w*/*v* yeast extract (BD, Sparks, MD, USA), 1.0% *w*/*v* sodium chloride (Merck, Darmstadt, Germany), and 1.0% *w/v* agar (BD, Sparks, MD, USA). Plates were inverted and incubated at 37 °C for 24 h. Posteriorly, the agar was removed and stained with 2% *w*/*v* crystal violet (Laborclin, Pinhais, PR, Brazil) for 2 h [24]. The motility halo was measured to the nearest millimeter. As a negative control, each isolate was inoculated in tryptone soy agar (BD, Sparks, MD, USA) under the same conditions.

#### 2.5.5. Rhamnolipids

*P. aeruginosa* strains were cultured in the presence (1/2, 1/4, and 1/8 MIC) or absence of bio-AgNPs in LB broth (Neogen, Lansing, MI, USA) at 37 °C for 24 h. Then, 10 µL of inoculum was placed at the center of a cetyltrimethylammonium bromide (CTAB) agar plate containing 0.09% *w/v* monobasic potassium phosphate (Synth, Diadema, SP, Brazil), 0.11% *w/v* bibasic sodium phosphate (Synth, Diadema, SP, Brazil), 0.25% *w/v* sodium nitrate (Synth, Diadema, SP, Brazil), 0.01% *w/v* calcium chloride (Synth, Diadema, SP, Brazil), 0.04% *w/v* magnesium sulfate (Synth, Diadema, SP, Brazil), 0.2% *w/v* CTAB (Sigma–Aldrich, Steinheim am Albuch, Germany), 0.005% *w/v* methylene blue (Synth, Diadema, SP, Brazil), 0.5% *w/v* glucose (Synth, Diadema, SP, Brazil), and 2.0% *w/v* agar (Acumedia, Lansing, MI, USA). Plates were incubated at 37 °C for 48 h [25]. Rhamnolipid production was determined by measuring the halo of blue precipitate surrounding colonies.

#### 2.5.6. Alkaline Protease

*P. aeruginosa* strains were grown in LB broth (Neogen, Lansing, MI, USA) at 37 °C for 24 h in the presence (1/2, 1/4, and 1/8 MIC) or absence of bio-AgNPs. Then, 10 µL of supernatant from treated and untreated *P. aeruginosa* PAO1 and PA14 was added to a milk agar plate (pH 10.0) containing 1.0% *w/v* milk powder (Acumedia, Lansing, MI, USA), 0.1% *w/v* peptone (Acumedia, Lansing, MI, USA), 0.5% *w/v* NaCl (Synth, Diadema, SP, Brazil), and 2.0% *w/v* agar (Acumedia, Lansing, MI, USA) and was incubated at 37 °C for 24 h. Alkaline protease production was indicated by the formation of a clear halo around colonies. Halo diameters were measured and compared with those of the control [26].

#### 2.5.7. Elastase B (LasB)

The elastin–Congo Red (ECR) method was used to investigate LasB activity [27]. *P. aeruginosa* was cultured in LB broth (Neogen, Lansing, MI, USA) at 37 °C for 24 h in the presence (1/2, 1/4, and 1/8 MIC) or absence of bio-AgNPs. After centrifugation (8000 rpm, 10 min), 500 µL of the supernatant was added to 500 µL of 100 mM Tris-HCl buffer, pH 7.5, containing 10 mg of ECR (Sigma-Aldrich, Steinheim am Albuch, Germany). The mixture was incubated at 37 °C for 6 h under stirring (120 rpm). After centrifugation (8000 rpm, 10 min), the absorbance was measured at 495 nm on a Thermo Scientific Multiskan GO spectrophotometer.

#### 2.5.8. Pyocyanin

A liquid medium consisting of 2.0% *w/v* peptone (Neogen, Lansing, MI, USA), 0.14% *w/v* magnesium chloride (Synth, Diadema, SP, Brazil), and 1.0% *w/v* magnesium sulfate (Synth, Diadema, SP, Brazil) was used to assess pyocyanin production, as described by El-Mowafy et al. [28]. Briefly, 1 mL of a 0.5 McFarland-equivalent suspension of *P. aeruginosa* grown in LB broth (Neogen, Lansing, MI, USA) was inoculated in 30 mL of liquid medium in the presence (1/2, 1/4, or 1/8 MIC) or absence of bio-AgNPs. Cells were incubated at 37 °C for 24 h. Then, cultures were centrifuged, and 7.5 mL of the supernatant was transferred to a tube containing 4.5 mL of chloroform (Merck, Steinheim am Albuch, Germany). This mixture was vigorously homogenized on a vortex mixer for 20 s. The organic phase was collected (3 mL), acidified with 1.5 mL of 0.2 M hydrochloric acid, homogenized for 20 s, and centrifuged at 4600 rpm for 10 min. The absorbance of the resulting pink-colored solution was measured at 520 nm on a SPECORD S600 UV/VIS spectrophotometer (Analytica Jena, Germany). Pyocyanin concentrations were converted to µg/mL, by multiplying the optical density at 520 nm by 17,072 × 1.5. 

### 2.6. RNA Extraction and Real-Time Polymerase Chain Reaction (RT-qPCR)

For analysis of the expression of QS-related genes, total RNA was extracted from *P. aeruginosa* PAO1 and PA14 cells grown in the absence (reference) or presence of bio-AgNPs (1/2 MIC). Extraction was performed using TRIzol reagent (Invitrogen, Waltham, MA, USA) and the RNeasy Mini kit (QIAGEN, Germantown, MA, EUA ). DNA contamination was eliminated by using the RQ1-DNAse kit (Promega, USA). The quality of extracted RNA was verified by agarose gel electrophoresis and calculation of the A260/A280 ratio. Gene sequences were obtained from GenBank, and primers were designed and analyzed using Primer-BLAST and BioEdit Sequence Alignment Editor. Primer sequences are listed in Table 1. RT-qPCR was performed using the QuantiNova SYBR Green RT-PCR kit (QIAGEN, Germantown, MA, EUA) in a final volume of 20 µL, consisting of 10 µL of SYBR Green RT-PCR Master Mix, 0.2 µL of RT mix, 1 µL (20 µM) of each primer, 5 µL of bacterial RNA (50 ng/μL), and 2.8 µL of RNase-free water. Reactions were performed on a Rotor-Gene Q 2plex (QIAGEN, Germantown, MA, EUA) using the following steps: reverse transcription for 10 min at 50 °C, initial denaturation for 2 min at 95 °C, 40 cycles of 95 °C for 5 s, and hybridization and extension at 60 °C for 10 s. The *proC* gene was selected as an internal control and was used to normalize the expression of target genes.

For analysis of the expression of QS-related genes, total RNA was extracted from *P. aeruginosa* PAO1 and PA14 cells grown in the absence (reference) or presence of bio-AgNPs (1/2 MIC). Extraction was performed using TRIzol reagent (Invitrogen, Waltham, MA, USA) and the RNeasy Mini kit (QIAGEN, Germantown, MA, EUA ). DNA contamination was eliminated by using the RQ1-DNAse kit (Promega, USA). The quality of extracted RNA was verified by agarose gel electrophoresis and calculation of the A260/A280 ratio. Gene sequences were obtained from GenBank, and primers were designed and analyzed using Primer-BLAST and BioEdit Sequence Alignment Editor. Primer sequences are listed in Table 1. RT-qPCR was performed using the QuantiNova SYBR Green RT-PCR kit (QIAGEN, Germantown, MA, EUA) in a final volume of 20 µL, consisting of 10 µL of SYBR Green RT-PCR Master Mix, 0.2 µL of RT mix, 1 µL (20 µM) of each primer, 5 µL of bacterial RNA (50 ng/μL), and 2.8 µL of RNase-free water. Reactions were performed on a Rotor-Gene Q 2plex (QIAGEN, Germantown, MA, EUA) using the following steps: reverse transcription for 10 min at 50 °C, initial denaturation for 2 min at 95 °C, 40 cycles of 95 °C for 5 s, and hybridization and extension at 60 °C for 10 s. The *proC* gene was selected as an internal control and was used to normalize the expression of target genes.

### 2.7. Statistical Analysis

Statistical analysis was performed using R Studio version 1.2.5001 (R, Boston, MA, USA), and graphs were constructed using GraphPad Prism version 8.4.2 (GraphPad Software Inc., San Diego, CA, USA). Values of *p* < 0.05 were considered significant. Data were subjected to analysis of variance followed by Tukey’s test. For RT-qPCR data, randomization tests were conducted using REST software version 2.0.13 (Qiagen, Hilden, Germany) to identify statistically significant differences between groups.

## 3. Results

### 3.1. MIC of bio-AgNPs against P. aeruginosa

**Figure 1:**
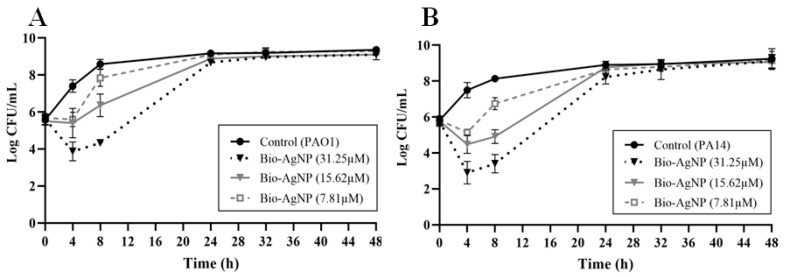
Growth curve of *Pseudomonas aeruginosa.* (**A**) PAO1 and (**B**) PA14 in the presence or absence (control) of subinhibitory concentrations (31.25 µM, 15.62 µM, and 7.81 µM) of biogenic silver nanoparticles (Bio-AgNP). Bacteria were inoculated at a density of 5 × 10^5^ CFU/mL. Results are expressed as mean ± standard deviation.

MIC values were determined by using bio-AgNPs at different concentrations (7.81 to 1000 µM). MIC values for *P. aeruginosa* PAO1 and PA14 were 62.5 µM. Bio-AgNPs were also tested at subinhibitory concentrations, 1/2 (31.25 µM), 1/4 (15.62 µM), and 1/8 MIC (7.81 µM), to evaluate whether treatments would have any effect on PAO1 and PA14 growth. As shown in Figure 1A,B, PAO1 and PA14 growth was not influenced (*p* > 0.05) by treatment with bio-AgNPs at subinhibitory concentrations after 24 and 48 h.

### 3.2. Effect of bio-AgNPs on P. aeruginosa Virulence Factors

We investigated the effects of bio-AgNPs at subinhibitory concentrations (7.81 µM, 15.62 µM, and 31.25 µM) on QS-controlled virulence factors, such as biofilm formation, motility, and rhamnolipid, alkaline protease, LasB, and pyocyanin production.

#### 3.2.1. Biofilm Formation

The crystal violet method revealed no significant change in biofilm formation in PAO1 after treatment with bio-AgNPs (Table 2). However, all three bio-AgNP treatments significantly increased (*p* < 0.05) biofilm formation in PA14, by 41.22% to 127.86%.

#### 3.2.2. Effect on Swarming, Swimming, and Twitching Motilities

**Figure 2:**
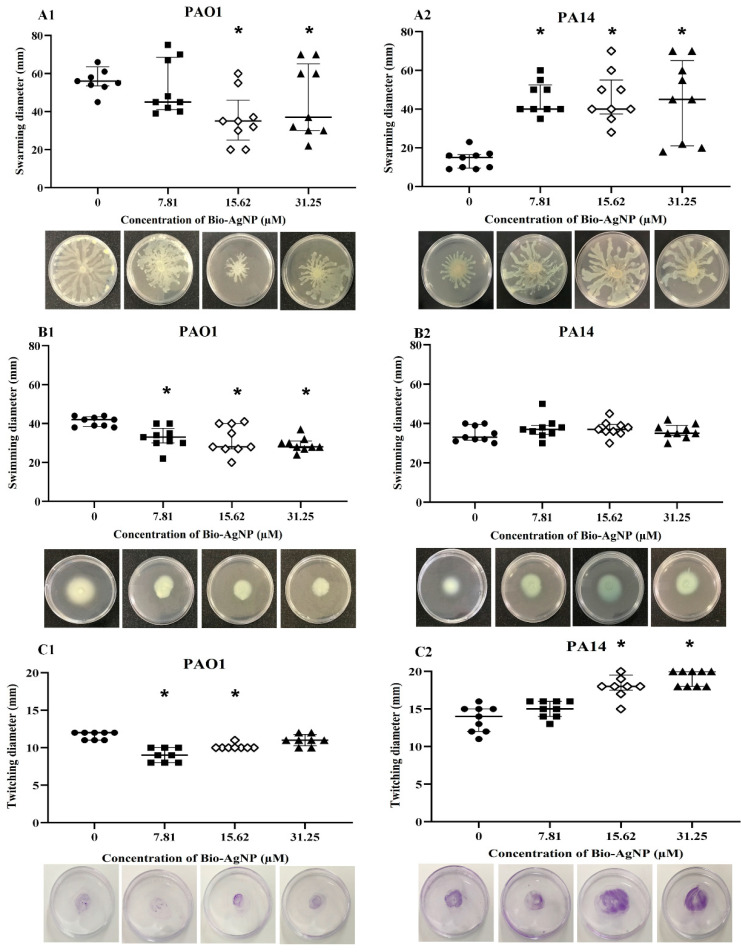
Effect of bio-AgNPs on swarming, swimming, and twitching motilities in *Pseudomonas aeruginosa* PAO1 and PA14. Swarming motility in (**A1**) PAO1 and (**A2**) PA14. Swimming motility in (**B1**) PAO1 and (**B2**) PA14. Twitching motility in (**C1**) PAO1 and (**C2**) PA14. Each data point represents the result of one treatment, and the mean of three replications is indicated by a black bar. Images below the graphs are representative photographs of PAO1 and PA14 motility halos. Asterisks indicate significant differences between means and the control at *p* < 0.05 by analysis of variance followed by Tukey’s test.

In the present study, the effects of sub-MIC levels of bio-AgNPs on the swarming, swimming, and twitching motilities in *P. aeruginosa* were investigated by determining the diameter (mm) of the motility halo. As depicted in Figure 2A1, bio-AgNPs inhibited the swarming motility in PAO1. After treatment with 31.25 µM bio-AgNPs, the mean diameter of the motility halo was 45.67 mm, representing an inhibition rate of 22.45% compared with the untreated control (58.89 mm diameter). In PA14, bio-AgNPs induced a significant increase (*p* < 0.05) in the swarming motility (Figure 2A2). The diameter of the swarming motility halo increased from 13.89 mm (untreated control) to 45.00–45.89 mm, representing an increase of up to 230.40%.

Similarly, PAO1 swimming motility was significantly (*p* < 0.05) inhibited by bio-AgNP treatments (Figure 2B1). The control exhibited a motility halo of 41.00 mm in diameter, whereas treated bacteria had a swimming motility halo of 29.33 to 32.78 mm, with a reduction of 20.05% to 28.46%. In PA14, treatments did not influence the swimming motility (Figure 2B2).

PAO1 twitching motility was significantly inhibited (*p* < 0.05) by treatment with 7.81 and 15.62 µM bio-AgNPs, leading to reductions of 22.58% and 12.90%, respectively (Figure 2C1). By contrast, in PA14, the twitching motility increased by 34.15% and 39.84% (*p* < 0.05) after treatment with 15.62 and 31.25 µM bio-AgNPs, respectively (Figure 2C2).

#### 3.2.3. Rhamnolipids

**Figure 3:**
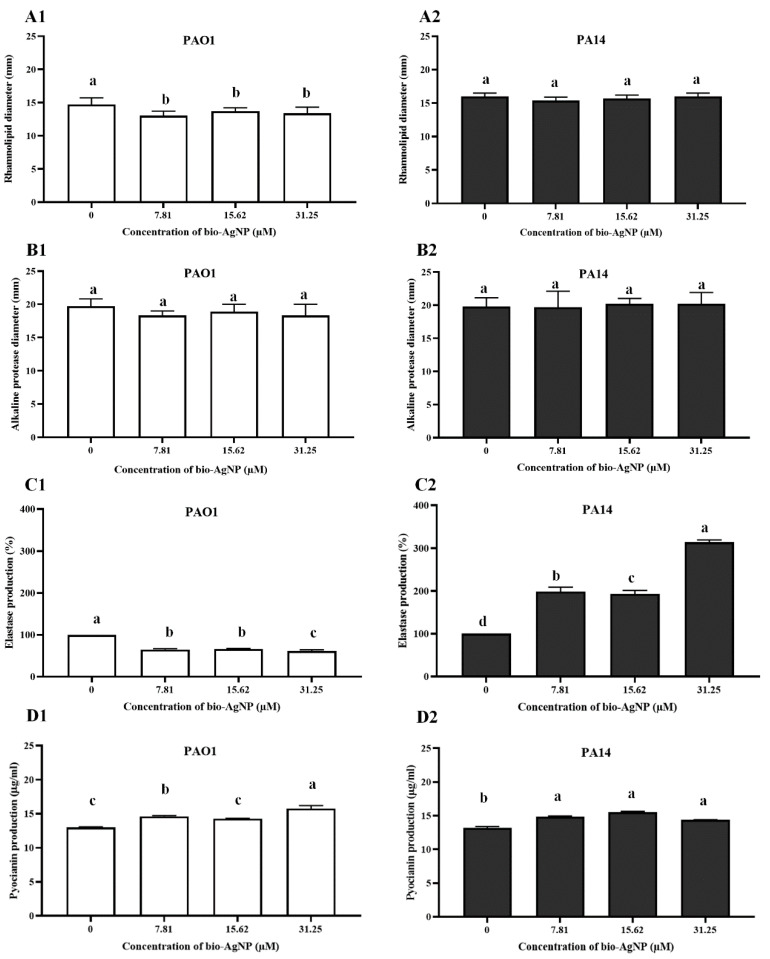
Effect of bio-AgNPs on virulence factors of *Pseudomonas aeruginosa* PAO1 and PA14. Rhamnolipid production in (**A1**) PAO1 and (**A2**) PA14. Alkaline protease production in (**B1**) PAO1 and (**B2**) PA14. Elastase B production in (**C1**) PAO1 and (**C2**) PA14. Pyocyanin production in (**D1**) PAO1 and (**D2**) PA14. Results are expressed as mean ± standard deviation (n = 3). a–c Tukey’s honestly significant difference test at *p* < 0.05.

All bio-AgNP treatments significantly reduced (*p* < 0.05) rhamnolipid production by PAO1 (Figure 3A1). However, treatments did not influence rhamnolipid production by PA14 (Figure 3A2).

#### 3.2.4. Alkaline Protease

Alkaline protease production was not affected by bio-AgNP treatments (Figure 3B1,B2). The mean halo diameters of PAO1 and PA14 were 19.67 and 19.78 mm, respectively, before treatment, and 18.33–18.89 and 19.67–20.22 mm, respectively, after treatment.

#### 3.2.5. LasB

Figure 3C depicts the LasB activity of strains as a function of bio-AgNP concentration. In PAO1, LasB activity decreased (*p* < 0.05) by 33.79%, 30.27%, and 39.01% with exposure to bio-AgNPs at 7.81, 15.62, and 31.25 µM, respectively (Figure 3C1). By contrast, LasB activity increased (*p* < 0.05) by 99.18%, 93.76%, and 214.74% in PA14 with exposure to 7.81, 15.62, and 31.25 µM bio-AgNPs, respectively (Figure 3C2).

#### 3.2.6. Pyocyanin

Pyocyanin production by *P. aeruginosa* in the presence of different concentrations of bio-AgNPs was determined spectrophotometrically at 520 nm. In both PAO1 and PA14, there was an increase in pyocyanin production with all treatments (*p* < 0.05). In the absence of bio-AgNPs, PAO1 and PA14 produced 13.51 and 14.74 µg/mL pyocyanin, respectively. PAO1 treated with 31.25 µM bio-AgNPs showed the highest pyocyanin production (16.16 µg/mL, representing an increase of 19.70%. In PA14, the highest pyocyanin production (16.39 µg/mL) was achieved by treatment with 31.25 µM bio-AgNPs, affording an increase of 11.21% (Figure 3D).

### 3.3. Gene Expression

**Figure 4:**
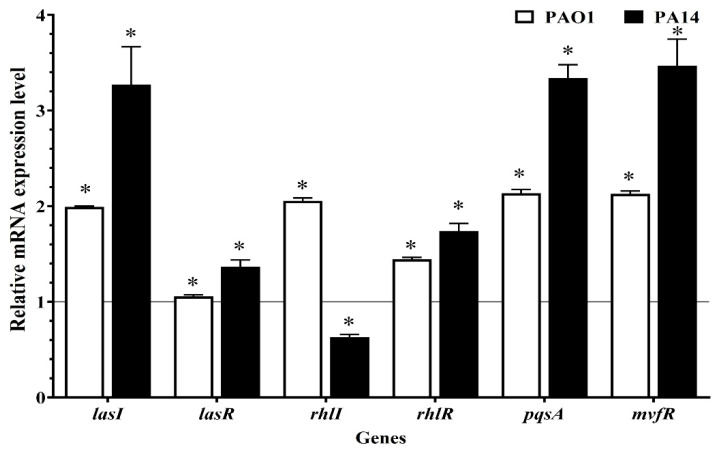
Relative expression of quorum-sensing regulatory genes in *Pseudomonas aeruginosa* treated with bio-AgNPs. The mean relative gene expression was normalized to that of the reference gene *proC*. * *p* < 0.05.

The relative expression of the six regulatory genes of the QS system (*lasI, lasR, rhlI, rhlR, pqsA*, and *mvfR*) of *P. aeruginosa* PAO1 and PA14 was assessed after 24 h of treatment with 31.25 µM bio-AgNPs, as shown in Figure 4. Gene expression was higher in *P. aeruginosa* PAO1 treated with bio-AgNP than in the control (*p* < 0.05): the expression of *lasI, lasR, rhlI, rhlR, pqsA*, and *mvfR* increased by 2.0-, 1.1-, 2.1-, 1.4-, 2.1-, and 2.1-fold, respectively. In PA14, the expression of *lasI, lasR, rhlR, pqsA*, and *mvfR* increased (*p* < 0.05) by 3.3-, 1.4-, 1.7-, 3.3-, and 3.5-fold, respectively, with bio-AgNP treatment. *rhlI* expression, however, was downregulated, being 0.6-fold lower in treated bacteria (*p* < 0.05).

## 4. Discussion

Given the need for advances in the control of multidrug-resistant bacteria and antivirulence therapeutic strategies, we investigated the potential of bio-AgNPs as bacterial control agents. Research efforts have been directed toward the use of nanotechnology and nanoparticles to target the QS system and/or virulence of microorganisms such as *P. aeruginosa* [16,29,30]. One of the advantages of using bio-AgNPs is related to their low cytotoxicity. Scandorieiro et al. [31] showed that this biogenic silver nanoparticle did not show cytotoxicity at concentrations up to 97.22 µM in human RBC and HEp-2 cells. In the present study, we used concentrations up to 31.25 µM of bio-AgNPs.

In the current study, we identified significant differences in phenotypic characteristics between the evaluated reference strains. In PAO1, the swarming, swimming, and twitching motilities, as well as rhamnolipid and elastase production, were significantly reduced in the presence of bio-AgNPs at sub-MIC levels. However, in PA14, bio-AgNP treatment significantly enhanced swarming motility, twitching motility, biofilm formation, and elastase and pyocyanin production.

The reference strains exhibit different genotypic characteristics. *P. aeruginosa* PAO1 is a moderately virulent strain belonging to a relatively rare clonal group (ST 549). PA14, on the other hand, is highly virulent and belongs to the most common clonal group (ST 253). Furthermore, PA14 has two pathogenicity islands (PAPI-1 and PAPI-2), which are absent in PAO1 [32,33,34] The strains also differ in genome size (6.3 Mbp in PAO1 and 6.5 Mbp in PA14) and have unique genomic regions (54 regions in PAO1 and 58 regions in PA14) [35]. Such factors could explain the different responses of strains to bio-AgNP treatment.

As evidenced by the biofilm formation assay, untreated PAO1 showed greater capacity for biofilm formation than untreated PA14. The reduced capacity of PA14 for biofilm formation might be related to mutation of the *ladS* gene, which has a deleterious effect on biofilm production [33]. Kasetty et al. [36] also showed that in microfluidic biofilm culture conditions, PAO1 quickly outcompetes PA14 in density. Furthermore, PA14 exhibits a competitive fitness advantage when invading a preformed biofilm and is better able to tolerate starvation than PAO1 in the biofilm context.

Surprisingly, after bio-AgNP treatment, PA14 exhibited a significant increase in biofilm formation. The mechanism of biofilm formation differs between PAO1 and PA14, given that each strain uses a different exopolysaccharide as the predominant structural biofilm component. Whereas PAO1 uses the Wsp system to produce Psl, PA14 uses the Pil-Chp system for Pel production [37,38]. Biofilm formation is dependent on several factors, such as synthesis of exopolysaccharides Psl and Pel, alginates, extracellular DNA, and adhesins (flagella and type IV pili). These factors contribute to maturation, antibiotic resistance, and biofilm persistence [39]. Exopolysaccharides are responsible for bacterial adhesion, biofilm formation, and architecture stability [40]. Extracellular DNA contributes to twitching motility and the supply of nutrients to bacterial cells [41].

It is important to highlight that there is an indirect link between biofilm formation and the QS system, related to the control of the swarming and twitching motilities as well as rhamnolipid [42] and pyocyanin [43] production. *P. aeruginosa* displays three types of motilities (swarming, swimming, and twitching), which allow surface colonization and exploration of new environments [44]. The first step in biofilm formation is bacterial fixation to surfaces via polar flagella and production of adhesion proteins, such as type IV pili, thereby allowing cells to spread to surrounding areas [45].

In the present study, the significant increase in PA14 swarming motility, twitching motility, and pyocyanin production with bio-AgNP treatment contributed to biofilm formation. According to Persat et al. [46], virulence factors can be activated by structural elements (e.g., type IV pili). *P. aeruginosa* uses these structures for surface recognition and activation of the Chp chemosensory system, which regulates cAMP (second messenger) and induces virulence gene transcription. Therefore, the expressive increase in the swarming and twitching motilities in PA14, mediated by type IV pili, might have favored the increase in other virulence factors.

Ouyang et al. [30] treated *Pseudomonas putida* KT2440 with low concentrations of zinc nanoparticles (0.5–30 mg/L) and observed an increase in biofilm formation. Protein and sugar contents of biofilm also increased with treatment. Garuglieri et al. [47] reported that subinhibitory concentrations of AgNPs (0.01 µg/mL) might increase the swimming motility in *Escherichia coli*. In the study of Saeki et al. [48], *P. aeruginosa* isolates from clinical and environmental sources were found to exhibit increased swarming, swimming, and twitching motilities following treatment with bio-AgNPs. In such cases, bio-AgNPs possibly stimulated a response to stress induced by environmental conditions, as described by Villa et al. [49].

PAO1 showed a significant reduction in LasB production with bio-AgNP treatment, and the opposite was observed in PA14 (*p* < 0.05). LasB, also known as pseudolysin, is associated with vascular inflammation in *P. aeruginosa* infections [50]. In agreement with the present study, in which PA14 showed increased biofilm formation with bio-AgNP treatment, Yu et al. [51] demonstrated that LasB is crucial for biofilm formation. The authors found that ΔlasB mutant *P. aeruginosa* has reduced capacity for biofilm formation owing to a decrease in rhamnolipid synthesis.

The alkaline protease assay revealed no differences in protease production with bio-AgNP treatment in either PAO1 or PA14. Alkaline protease causes severe damage to host tissues by disruption of cytoskeleton structures and degradation of fibronectin and laminin, important components of the endothelium [50]. Both alkaline protease and LasB are able to inhibit neutrophil function, interfering with chemotaxis. As a result, bacteria gain an advantage by escaping phagocytes of the host defense system [52].

To further understand bio-AgNP at the molecular level and to lend support to the outcome of our in vitro results, we followed up with qRT-PCR analysis. *lasI, rhlI,* and *pqsA* expression is necessary for the synthesis of QS signaling molecules in *P. aeruginosa*. Thus, the transcriptional regulators lasR, rhlR, and mvfR were analyzed in this study. Although most PAO1 virulence factors decreased after bio-AgNP treatment, the respective genes were upregulated in both strains (PAO1 and PA14). Bio-AgNP-treated PAO1 bacteria exhibited a higher expression (upregulation) of the evaluated genes compared with the control (*p* < 0.05). In PA14, only rhlI was downregulated; the other genes were more highly expressed (*p* < 0.05) than in the control.

Typically, the common mechanisms underlying quorum-sensing interference include inhibition of signal biosynthesis, signal degradation, and interruption of the reception signal molecules [53]. Based on our results, the possible mechanism underlying the action of bio-AgNPs involves the induction of the rhl and/or pqs system of PAO1 and induction of the las and/or pqs system of PA14.

The las system is the master regulator of QS, as it induces the expression of both rhl and PQS pathways in *P. aeruginosa* [54]. The synthase proteins LasI and RhlI are responsible for the production of the autoinducers 3-oxo-C12-AHL and C4-AHL, respectively. When the concentration of such molecules reaches a given threshold, 3-oxo-C12-AHL binds to the receptor lasR, thereby inducing the expression of virulence factors, such as LasB, exotoxin, and proteases, and activating the rhlI/R system. The autoinducer C4-AHL binds to rhlR and controls motility and expression of genes that encode LasB, pyocyanin, and rhamnolipid. The third autoinducer, PQS, binds to the receptor mvfR, regulating pyocyanin expression and activating the rhlI system [14]. Therefore, the 2.0-fold increase in *pqsA* and *mvfR* gene expression in PAO1 and PA14 was probably associated with increased pyocyanin production resulting from bio-AgNP treatment; the 0.6-fold reduction in *rhlI* expression in PA14, however, did not lead to a decrease in virulence in phenotypic assays.

Liao et al. [55] demonstrated that the main mechanism of action of AgNPs against multidrug-resistant isolates of *P. aeruginosa* involves the imbalance of oxidation processes and impairment of the elimination of oxygen reactive species. Cugini et al. [56] proposed that compounds such as farnesol, which promote reactive oxygen species production, may enhance the expression of the PQS system by activating rhlR in *P. aeruginosa*.

The decrease in motility and rhamnolipid and LasB production in PAO1 might be explained by the effect of bio-AgNPs on *lasR* expression: expression increased 1.1-fold. Given the interconnection of QS systems and the fact that lasR controls the activation of connected cascades [57], it is suggested that the effect of bio-AgNP treatment on *lasR* expression was not sufficient to induce a phenotypic increase in virulence factors. By contrast, in PA14, the 1.4-fold increase in *lasR* expression resulted in increased motility and LasB production.

Our findings were similar to those of previous studies investigating the influence of AgNPs on the QS system of *P. aeruginosa*. Yang and Alvarez [58] observed that subinhibitory concentrations of AgNPs (21.6 and 108 µg/L) could induce the QS system (*lasI, lasR, rhll*, and *rhlR* expression) in PAO1 and increase biofilm formation. Li et al. [29] studied several nanomaterials (e.g., silver, iron, zinc oxide, graphene) and found that AgNPs (100 µg/L) enhanced 3-oxo-C12-AHL synthesis, protease production, biofilm formation, and *lasR* expression in PAO1. Ouyang et al. [30], in assessing the effect of low concentrations of zinc nanoparticles on *P. putida*, observed an increase in the expression of QS regulatory genes with treatment.

Previous studies have found that AgNP treatment affected virulence factor production, and QS. Singh et al. [59] reported that AgNPs (28 nm) synthesized by *Rhizopus arrhizus* BRS-07 inhibited the expression of QS regulatory genes (*lasI, lasR, rhlI*, *rhlR*, and *fabH2*) in *P. aeruginosa* PAO1. Liu et al. [60] combined AgNPs with 4-nitropyridine N-oxide for inhibition of biofilm formation and QS gene expression (*lasI, lasR, rhlI, rhlR, pqsA*, and *pqsR*). In the current research, bio-AgNPs were synthesized by *F. oxysporum* and measured 73 nm in size. Liu et al. [60] used a combination of nanoparticles with 4-nitropyridine N-oxide, whereas we used bio-AgNPs only.

Given that QS inhibition has shown high potential as an antivirulence strategy [6,9], understanding the mechanisms of pathogenicity in *P. aeruginosa* is of extreme importance, to ensure that antivirulence compounds are applied efficiently. As highlighted by Mohanty et al. [61], the effects of metal nanoparticles on the QS system are highly dependent on bacterial species. Therefore, given the variability of bacterial responses to different metal compounds, this approach needs to be well-studied and scientifically proven with complementary molecular studies on reference strains and isolates obtained from different sources (environmental and clinical).

## 5. Conclusions

Our results suggest that bacterial exposure to low concentrations of bio-AgNPs may enhance the expression of QS regulatory genes in *P. aeruginosa*. This model indicates that the target compound (bio-AgNP) affected functional genes involved in the biofilms’ formation and virulence production, mainly in PA14. This implies that the presence or accidental release of low concentrations of AgNPs may cause ecological imbalances, possibly leading to an increase in *P. aeruginosa* virulence. Thus, the new concept of antivirulence therapy needs to be carefully studied. Understanding bacterial virulence and pathogenicity mechanisms, which depend on a variety of extracellular and cell-associated factors, is essential for the development of potential targets for antivirulence therapy.

## Figures and Tables

**Table 1:** List of genes and their respective primers used in the RT-qPCR assay.

Target Gene	Primer	Sequence	Amplicon Size (bp)
*proC*	F	5′-GAG CAA CTG ATC GTC TCC ATC-3′	100
	R	5′-GGG TGT TGG GCA TGC AG-3′	
*lasI*	F	5′-GTA GGC GTG GAG AAG ATG ATG-3′	122
	R	5′-ATC TGG GTC TTG GCA TTG AG-3′	
*lasR*	F	5′-CTG TGG ATG CTC AAG GAC TAC-3′	111
	R	5′-CCA CTG CAA CAC TTC CTT CT-3′	
*rhlI*	F	5′-GTC GGT CTG GGA GCT TTC-3′	100
	R	5′-CAG GTA CCA GGC GCA TT-3′	
*rhlR*	F	5′-CTG TGG TGG GAC GGT TTG-3′	139
	R	5′-GGG TGA AGG GAA TCG TGT G-3′	
*pqsA*	F	5′-GCT GTA TTC GAT TCC CAA GAT G-3′	100
	R	5′-CCA GGT ATC GTC GAG CAG-3′	
*mvfR*	F	5′-GCT TCG CCT GAT CCC TTA C-3′	104
	R	5′-GCA GCA CCC GGA GAT TG-3′	

F: forward; R: reverse; pb: base pairs.

**Table 2:** Biofilm formation in *P. aeruginosa* after treatment with different subinhibitory concentrations of silver nanoparticles.

Strain	Compound	Concentration	Biofilm Formation ^1^	Inhibition Rate (%) ^2^	Increase Rate (%) ^2^
PAO1	Controle	0 µM	0.260 ± 0.040	-	-
PAO1	bio-AgNP	7.81 µM	0.257 ± 0.021	1.31%	-
PAO1	bio-AgNP	15.62 µM	0.256 ± 0.051	1.73%	-
PAO1	bio-AgNP	31.25 µM	0.238 ± 0.068	8.74%	-
PA14	Controle	0 µM	0.052 ± 0.007 ^c^	-	-
PA14	bio-AgNP	7.81 µM	0.118 ± 0.015 ^a^	-	127.86%
PA14	bio-AgNP	15.62 µM	0.085 ± 0.020 ^b^	-	64.09%
PA14	bio-AgNP	31.25 µM	0.073 ± 0.013 ^b^	-	41.22%

^1^ Values expressed in optical density (OD) 620 nm (mean ± standard deviation). ^2^ Inhibition rate or increase = (OD control group–OD experimental group)/OD control group. ^a–c^ Tukey’s test with significant difference *p* < 0.05. bio-AgNP: biogenic silver nanoparticles.

## Supplementary Materials

The following supporting information can be downloaded at: https://www.mdpi.com/article/10.3390/microorganisms10091755/s1, Table S1: Comparison of biofilm formation methods.
