# Supplementary material for: Effect of Biogenic Silver Nanoparticles on the Quorum-Sensing System of Pseudomonas aeruginosa PAO1 and PA14
Source: Microorganisms. 2022 Aug 30;10(9):1755. doi: 10.3390/microorganisms10091755 (PMC9504124; doi:10.3390/microorganisms10091755)
Supplement: Supplementary file 1 [file microorganisms-10-01755-s001.zip › microorganisms-1850931-supplementary.pdf]

Table S1. Comparison of biofilm formation methods.

This Work	Ramos-Vivas et al. [1]
Bacterial isolates were cultured on LB agar at 37 °C for 24 h.	Bacterial isolates were cultured on LB agar at 37 °C for 24 h with shaking
To each well were added 180 μL of LB broth and 20 μL of *P. aeruginosa* inoculum and incubated at 37 °C for 24 h.	Twenty-fve microliters were placed in each well containing 1.5 mL of culture medium. The plates were incubated for 48 h at 37 °C in static.
Each well was discarded and the cell layer washed three times with phosphate buffer solution (PBS, pH 7.2)	Planktonic cells were removed and wells containing bioflms were rinsed three times with distilled water
Fixed with 250 μL of absolute methanol PA for 10 min	-
Stained with a 1.0 % *w/v* aqueous solution of crystal violet for 15 min.	The remaining adherent bacteria were stained with 2 mL/well of crystal violet (0.7%) for 12 min.
Each well washed three times with ultrapure water.	Excess stain was removed by washes with distilled water.
Treated with 250 μL of 33% *v/v* glacial acetic acid.	Treated with 33% *v/v* glacial acetic acid.
Absorbance readings were taken at 620 nm.	Absorbance readings were taken at 620 nm.

Reference

1. Ramos-Vivas, J.; Chapartegui-González, I.; Fernández-Martínez, M.; González-Rico, C.; Fortún, J.; et al. Biofilm formation by multidrug resistant Enterobacteriaceae strains isolated from solid organ transplant recipients. *Sci. Rep.* **2019**, *9*, 8928.
